# Rat Group IIA Secreted Phospholipase A_2_ Binds to Cytochrome c Oxidase and Inhibits Its Activity: A Possible Episode in the Development of Alzheimer’s Disease

**DOI:** 10.3390/ijms232012368

**Published:** 2022-10-15

**Authors:** Adrijan Ivanušec, Jernej Šribar, Adrijana Leonardi, Maja Zorović, Marko Živin, Igor Križaj

**Affiliations:** 1 Department of Molecular and Biomedical Sciences, Jožef Stefan Institute, SI-1000 Ljubljana, Slovenia; 2 Doctoral School, Faculty of Medicine, University of Ljubljana, SI-1000 Ljubljana, Slovenia; 3 Brain Research Laboratory, Institute of Pathophysiology, Medical Faculty, University of Ljubljana, SI-1000 Ljubljana, Slovenia

**Keywords:** group IIA secreted phospholipase A_2_, receptor, mitochondrion, Alzheimer’s disease, β-neurotoxicity, ammodytoxin, snake venom, *Vipera ammodytes*

## Abstract

Alzheimer’s disease (AD), a progressive form of dementia, is characterized by the increased expression of secreted phospholipase A_2_ group IIA (GIIA) in the affected tissue and the dysfunction of neuronal mitochondria, similar to that induced by an orthologous GIIA from snake venom, β-neurotoxic ammodytoxin (Atx), in the motor neurons. To advance our knowledge about the role of GIIA in AD, we studied the effect of rat GIIA on the neuronal mitochondria and compared it with that of the Atx. We produced recombinant rat GIIA (rGIIA) and its enzymatically inactive mutant, rGIIA(D49S), and demonstrated that they interact with the subunit II of cytochrome c oxidase (CCOX-II) as Atx. rGIIA and rGIIA(D49S) bound to this essential constituent of the respiratory chain complex with an approximately 100-fold lower affinity than Atx; nevertheless, both rGIIA molecules potently inhibited the CCOX activity in the isolated rat mitochondria. Like Atx, rGIIA was able to reach the mitochondria in the PC12 cells from the extracellular space, independent of its enzymatic activity. Consistently, the inhibition of the CCOX activity in the intact PC12 cells and in the rat’s brain tissue sections was clearly demonstrated using rGIIA(D49S). Our results show that the effects of mammalian and snake venom β-neurotoxic GIIA on the neuronal mitochondria have similar molecular backgrounds. They suggest that the elevated extracellular concentration of GIIA in the AD tissue drives the translocation of this enzyme into local neurons and their mitochondria to inhibit the activity of the CCOX in the respiratory chain. Consequently, the process of oxidative phosphorylation in the neurons is attenuated, eventually leading to their degeneration. Atx was thus revealed as a valuable molecular tool for further investigations of the role of GIIA in AD.

## 1. Introduction

The secreted phospholipases A_2_ (sPLA_2_s) (EC 3.1.1.4) are physiologically and pathologically very important proteins [1,2]. In mammals, 11 structurally diverse groups (G) of sPLA_2_s exist: IB, IIA, IIC, IID, IIE, IIF, III, V, X, XIIA and XIIB [2]. One of the most studied is GIIA. It is often referred to as ‘inflammatory’ sPLA_2_ due to its important role in inflammation [3]. This protein also represents a key element of the host’s defense system—it protects the organism against Gram-positive bacteria, viral infections and infection from the malaria pathogen *Plasmodium falciparum*. The involvement of GIIA in several other processes has been indicated, most recently in the shaping of gut microbiota, with implications for systemic events, including allergy, immunity and cancer in proximal and distal tissues [4]. However, in the context of our research, the most relevant is its association with the aetiology of neurodegenerative diseases. This enzyme is involved in the regulation of neurotransmission and neuritogenesis [5]. In connection with the latter, endogenous GIIA has been detected in mitochondria [6], but its function in this organelle remains speculative. In a normal physiological situation, it does no harm to mitochondria, but when it is deregulated in some acute and chronic neurological diseases, degeneration occurs [7]. An example of this is the brains (hippocampi) of patients with Alzheimer’s disease (AD), in which significantly higher levels of GIIA mRNA, as well as the mature form of the enzyme, were found than in the brains of non-demented people [8]. GIIA secreted by reactive astrocytes around the amyloid plaques in an AD brain has been shown to regulate the processing of the amyloid precursor protein in neuronal cells [9,10]; however, no other clear evidence about the involvement of GIIA in AD is currently available. Mitochondrial dysfunctions are central players in AD [11]. In AD brains, the mitochondria degenerate, as they do when nerve cells are intoxicated by the presynaptic neurotoxic snake venom sPLA_2_s (β-neurotoxins). Apparently, an excessive extracellular GIIA affects the neurons in a similar way as the orthologous neurotoxic snake venom sPLA_2_s [12]. Describing how β-neurotoxins target and affect the neuronal mitochondria could advance the study of the role of endogenous GIIA in neurodegenerative diseases. This would eventually lead to new diagnostic and therapeutic solutions. Studies with ammodytoxin (Atx), a prototype β-neurotoxin from the venom of the nose-horned viper (*Vipera ammodytes ammodytes*), revealed the subunit II of cytochrome c oxidase (CCOX-II) in the respiratory chain as its specific mitochondrial receptor [13]. Consequently, it inhibited CCOX activity. As has just been shown, Atx is transported from the extracellular space to the neuronal mitochondria via a specific pathway, without the need to be phospholipolytically active [14].

To gain new insights into the potential function and dysfunction of the orthologous mammalian GIIA in mitochondria, we produced recombinant rat GIIA (rGIIA) and its enzymatically inactive mutant rGIIA(D49S) and investigated their effect on neuronal mitochondria. We showed that rGIIA and rGIIA(D49S) bind to the same site on CCOX-II as the β-neurotoxic Atx, but with a lower affinity. Nevertheless, their inhibition of CCOX activity was even more efficient than that of Atx. Like Atx, the rGIIA molecule was also able to reach the mitochondria in the PC12 cells from the extracellular space. Our results support the hypothesis that the elevated extracellular concentration of GIIA in AD brains induces the translocation of the enzyme into the neuronal mitochondria, where it acts in a similar way to the β-neurotoxic snake venom sPLA_2_ by inhibiting the process of oxidative phosphorylation. Atx can therefore serve as a tool for studying the molecular pathology of GIIA in AD. AD has no cure, mainly because it is usually diagnosed too late. Early diagnostic biomarkers are crucial, and GIIA is a good candidate.

## 2. Results

### 2.1. Recombinant rGIIA and rGIIA(D49S) Are Functional

The recombinant rat sPLA_2_ proteins, rGIIA and its enzymatically inactive mutant, rGIIA(D49S), were produced in *Escherichia coli* in an inactive form of the inclusion bodies. These inclusion bodies were isolated and dissolved, and the proteins were refolded in vitro. rGIIA and rGIIA(D49S) were purified to homogeneity using RP-HPLC, as confirmed by an SDS-PAGE analysis, N-terminal protein sequencing and mass spectrometry. The molecular mass of each product was identical to its theoretical mass, minus 14 Da, which confirmed that all seven disulfide bonds were formed. As the scrambling of disulfides in such a small protein is highly unlikely, we were confident that both of the sPLA_2_ molecules folded correctly. In the case of rGIIA, this could be confirmed by measuring its phospholipase activity on PyrPG vesicles. As expected, the phospholipase activity of rGIIA(D49S) was negligible due to the D49S point mutation in the catalytic site.

### 2.2. rGIIA Binds to the Same Mitochondrial Target as the Neurotoxic Atx

**Figure 1:**
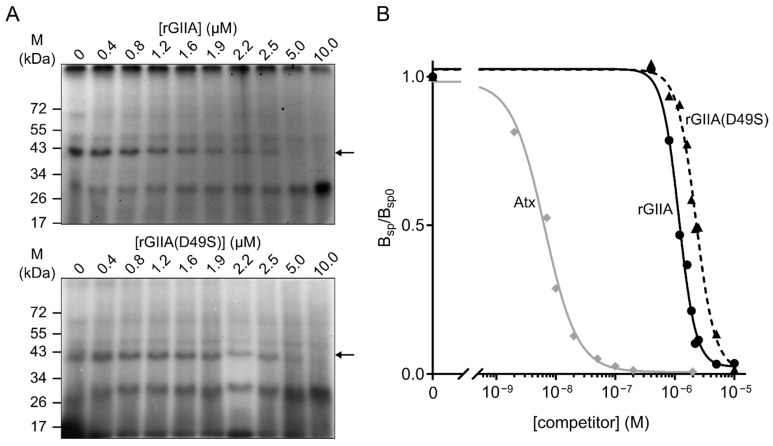
Heterologous competition assay revealed that rGIIA and its enzymatically inactive mutant rGIIA(D49S) inhibit ^125^I-Atx binding to its mitochondrial binding protein, CCOX-II. (**A**) Autoradiogram of the SDS-PAGE gel of detergent extracts of porcine cerebral cortex demyelinated P2 fraction labeled with ^125^I-Atx in the presence of the indicated concentrations of unlabeled sPLA_2_s, rGIIA or rGIIA(D49S). The unlabeled sPLA_2_s inhibited the formation of the specific adduct with an apparent molecular mass of 39 kDa (black arrows). (**B**) Dose–response curve relating the extent of cross-linking of ^125^I-Atx with the 25 kDa receptor to the concentration of unlabeled sPLA_2_s present during the incubation. The data were obtained by quantifying the intensity of the specific ^125^I-Atx adduct from the autoradiograms in (**A**).

We first tested whether rGIIA binds to the same mitochondrial target as Atx. From a porcine cerebral cortex, we isolated the P2d fraction of the mitochondrial membranes, which were previously revealed to be a rich source of this Atx receptor. A detergent extract of the membranes was incubated with the radio-iodinated Atx (^125^I-Atx) in the absence or presence of an excess of unlabeled sPLA_2_, rGIIA or rGIIA(D49S). The affinity-labeled extract was analyzed with an SDS-PAGE and the resulting gels were autoradiographed to observe the specific ^125^I-Atx–CCOX-II adduct at about 40 kDa (Figure 1A). Both rGIIA and rGIIA(D49S) inhibited the formation of this adduct, indicating that they both interfere with the binding of the Atx to its binding site on CCOX-II. The data used to generate dose–response curves (Figure 1B) were obtained by quantifying the intensity of the specific ^125^I-Atx–CCOX-II adduct formed in the presence of a given concentration of the competitor. From the curves, the half-maximum inhibitory concentrations (IC_50_) for rGIIA and rGIIA(D49S) were calculated to be 1.2 µM and 2.2 µM, respectively. Compared with the IC_50_ for Atx, which was 6 nM, these two values were about 100-fold higher. Although we showed in this experiment that rGIIA and its enzymatically inactivated form interfere with the binding of Atx to CCOX-II, we cannot claim yet that they also physically interact with this constituent of the CCOX complex.

### 2.3. rGIIA-Binding Protein in Mitochondria Is CCOX-II

**Figure 2:**
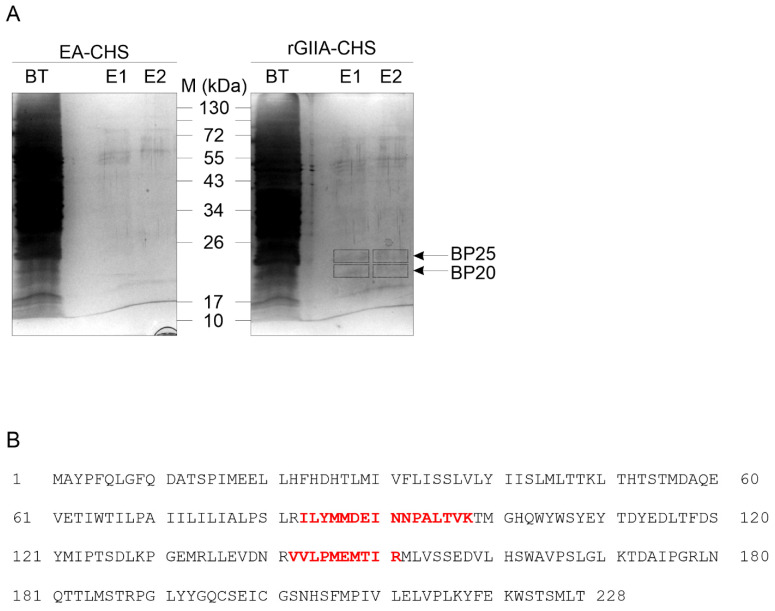
CCOX-II is a specific binding protein of rGIIA. This was confirmed when the CCOX-II was identified in the bound fraction of mitochondrial extract using the rGIIA-affinity media. (**A**) Porcine cerebral cortex P2d membranes were extracted with a detergent and the extract was chromatographed on an rGIIA-affinity column (rGIIA-CHS) and on a control column (EA-CHS). Proteins were eluted by lowering the pH from 7.4 to 5. The collected, unbound (BT) and bound fractions (E1 and E2) were analyzed with SDS-PAGE and the proteins were visualized via silver staining. Black boxes indicate the positions of excised bands. (**B**) The bands BP25 and BP20 were excised, and the proteins were reduced, alkylated and subjected to in-gel trypsin digestion. The resulting peptides were extracted from the gel and were analyzed by LC-MS/MS. Two tryptic peptides (red) from BP25 were identified as parts of the CCOX subunit II. No peptides could be identified in BP20.

To confirm the physical interaction between the rGIIA molecules and the CCOX-II, we immobilized the rGIIA on an affinity chromatography resin. The mitochondrial membranes in the P2d fraction of the porcine cerebral cortex were extracted with detergent and the soluble fraction was incubated with the rGIIA-CH-Sepharose (rGIIA-CHS) gel. After extensively washing the gel with a binding buffer (pH 7.4), the bound proteins were eluted by lowering the pH of the buffer by two units (pH 5.0). Specifically retained proteins were spotted by comparing the SDS-PAGE patterns of the proteins eluted from the rGIIA-CHS column and a mock column EA-CHS, where ethanolamine was used instead of rGIIA to bind the reactive groups on the CHS. Two specific protein bands were detected: one at an apparent molecular mass of 25 kDa and the other at 20 kDa (Figure 2A). Both were analyzed using tandem mass spectrometry (MS/MS). The 25 kDa band contained CCOX-II (Figure 2B), while no protein could be identified in the 20 kDa sample. We were thus able to demonstrate that rGIIA and CCOX-II physically interact. rGIIA has the binding site on CCOX-II, which overlaps with the binding site of the Atx. rGIIA apparently also binds to another mitochondrial protein that remains to be identified.

### 2.4. rGIIA Inhibits CCOX Activity in Isolated Mitochondria

**Figure 3:**
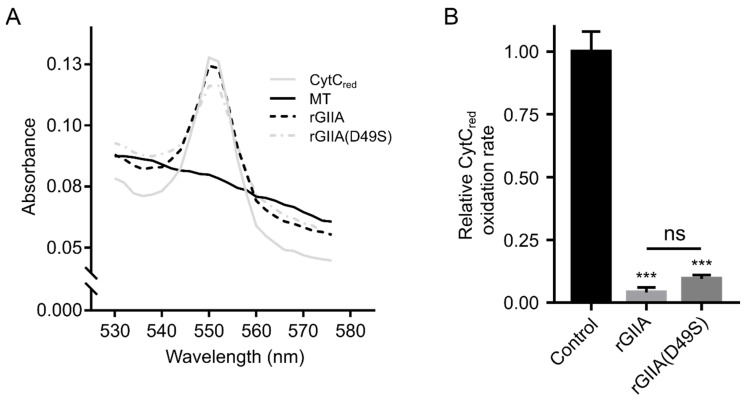
rGIIA or rGIIA(D49S) inhibits the enzyme activity of CCOX in isolated mitochondria. Mitochondria were isolated from PC12 cells and were incubated in either the absence (MT) or the presence of 1 μM rGIIA or 1 μM rGIIA(D49S). After the addition of the reduced cytochrome c (CytC_red_), which is a CCOX substrate, the change in absorbance at 550 nm (A_550_) (where CytC_red_ has maximum absorption) was measured. (**A**) Representative absorbance spectra of the samples measured 10 min after the addition of CytC_red_ in the range 530–576 nm. The inhibition of the CCOX enzymatic activity is indicated by retaining a high A_550_ compared with the positive control (MT). In the presence of both rGIIA and rGIIA(D49S), the CCOX activity was significantly inhibited. (**B**) The relative CytC_red_ oxidation rates were calculated as described in the Materials and Methods section. The results are presented as the means ± S.E.M. of at least three independent experiments and the statistical significance is indicated (*** *p* < 0.0001; and ns *p* > 0.05; one-way ANOVA with Tukey’s post-hoc test).

To answer the question about whether rGIIA also affects the activity of CCOX by binding to it, we performed a CCOX-activity assay on isolated mitochondria from the PC12 cells. Mitochondria were incubated at room temperature in the presence or absence of rGIIA or rGIIA(D49S), followed by the addition of the reduced form of cytochrome c (CytC_red_). The oxidation of CytC_red_ to CytC, a reaction catalyzed by CCOX, was then monitored by measuring the absorbance of the reaction mixture at 550 nm (A_550_), where CytC_red_ has a characteristic absorption maximum, whereas CytC does not (Figure 3A). The presence of 1 µM rGIIA in the suspension of mitochondria substantially reduced the rate of CytC_red_ oxidation (Figure 3B). The enzymatically inactive rGIIA(D49S) caused a very similar effect, as was already observed for Atx(D49S) [13].

The results showed that rGIIA, as its enzymatically inactive mutant, potently inhibited the activity of CCOX in isolated mitochondria. As in the case of Atx, this implies that the effect was due to the binding of the sPLA_2_ to the CCOX and not to its phospholipase activity.

### 2.5. rGIIA(D49S) Internalizes into PC12 Cells and Colocalizes with Mitochondria

**Figure 4:**
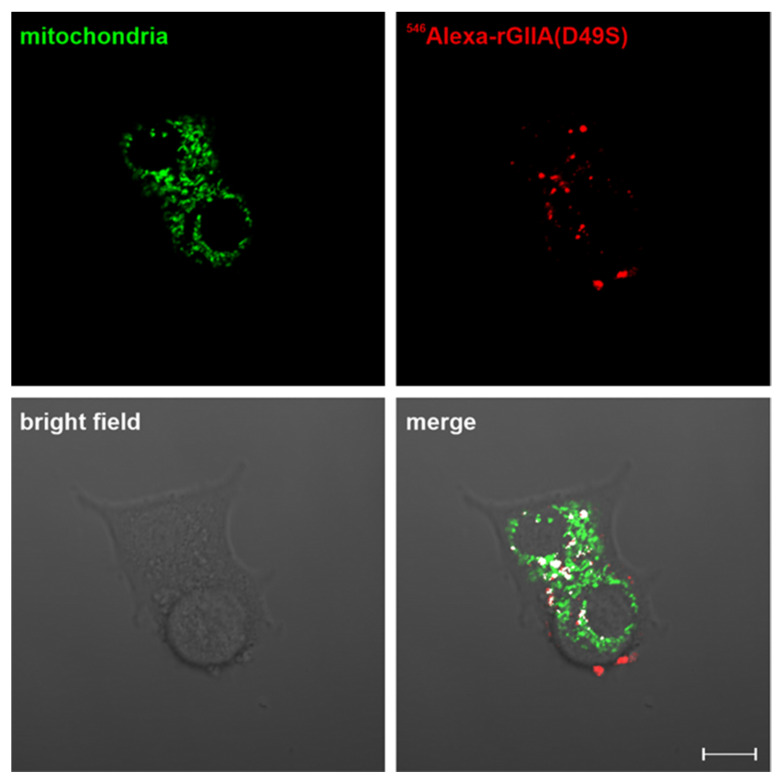
rGIIA can reach mitochondria in PC12 cells from the extracellular space independently of its enzymatic inactivity. PC12 cells were incubated with 100 nM ^546^Alexa-rGIIA(D49S) for 3 h and mitochondria were labeled with MitoTracker Green FM dye. Cells were then analyzed with confocal microscopy. Representative confocal fluorescence microscopy image shows ^546^Alexa-rGIIA(D49S) (red) and MitoTracker-labeled mitochondria (green); colocalization in merged image is shown in white. The extent of colocalization of ^546^Alexa-rGIIA(D49S) and mitochondria in PC12 cells, expressed in terms of the Manders’ coefficient, was 0.05 ± 0.01, mean ± S.E.M., as calculated from 7 images. Scale bar, 10 µm.

To investigate the biological relevance of the demonstrated interaction between rGIIA and CCOX-II, which caused the inhibition of the CCOX activity in isolated mitochondria, we tested whether the externally added rGIIA molecule could be internalized into the mitochondria of living PC12 cells. Like Atx, a snake venom orthologue of rGIIA, rGIIA was also able to reach the mitochondria of the PC12 cells from the media (Supplementary Figure S1). Just recently, it was reported that Atx was able to enter the mitochondria of the PC12 cells independently of its enzyme activity [14]; so, we also tested such an ability for the enzymatically inactive mutant of rGIIA, rGIIA(D49S). The mutant was fluorescently labeled with ^546^Alexa and added to the PC12 cells in a culture. Following the incubation, the mitochondria were counterstained and inspected under a confocal microscope. The red fluorescence of the ^546^Alexa-rGIIA(D49S) was spotted inside the cells, including in the mitochondria (Figure 4). The extent of the colocalization of the ^546^Alexa-rGIIA(D49S) and mitochondria in PC12 cells was 0.05 ± 0.01, expressed in terms of the Manders’ coefficient. From the results, we can conclude that the rGIIA molecule can enter the mitochondria from the extracellular milieu. An encounter between the extracellular rGIIA and the mitochondrial CCOX is thus possible, and its consequences are (patho)physiologically relevant.

To avoid any interference with the side effects possibly induced in the PC12 cells by the phospholipase activity of the rGIIA, we decided to perform all the experiments on cells, either PC12 cells or brain neurons, using only its enzymatically inactive variant, rGIIA(D49S).

### 2.6. rGIIA(D49S) Showed Only Minor Effect on Mitochondrial Membrane Potential in PC12 Cells

**Figure 5:**
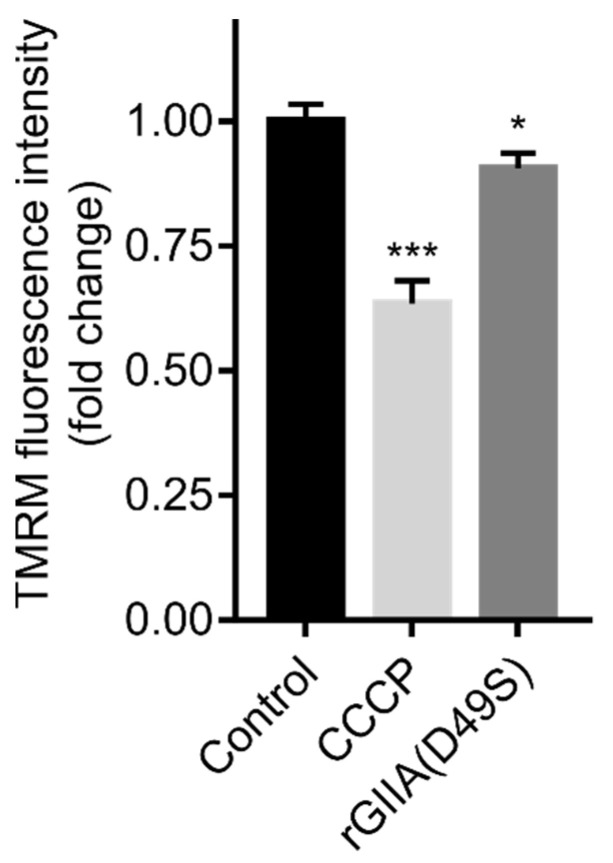
rGIIA(D49S) lowers TMRM (tetramethylrhodamine methyl ester) fluorescence intensity in PC12 cells. PC12 cells were starved overnight in the absence of serum and were then incubated for 3 h in the presence of either 1 µM rGIIA(D49S) or 100 µM carbonyl cyanide 3-chlorophenylhydrazone (CCCP), an uncoupler of mitochondrial membrane potential (MMP) that served as a positive control. Then, the cells were stained with TMRM dye and TMRM fluorescence intensity, an indicator of MMP, was evaluated using flow cytometry. Untreated cells served as a negative control. Results are presented as means ± SD of at least three independent experiments and results that were statistically significantly different from the control are indicated (* *p* < 0.05; *** *p* < 0.001; one-way ANOVA with Dunnett’s post-hoc test).

For the reason explained in Section 2.5., the effect on the tetramethylrhodamine methyl ester (TMRM) fluorescence intensity, an indicator of the mitochondrial membrane potential (MMP) in the PC12 cells, was only evaluated using rGIIA(D49S). The cells were incubated in the presence or absence of rGIIA(D49S). Then, the TMRM fluorescence intensity was measured and the cell death was assessed using flow cytometry. Compared with the untreated cells, rGIIA(D49S) slightly but significantly decreased the TMRM fluorescence intensity (Figure 5). Carbonyl cyanide 3-chlorophenylhydrazone (CCCP), a positive control that uncouples the MMP, caused a reduction in the TMRM fluorescence intensity of about 40%. Compared with the TMRM fluorescence intensity decrease with the positive control, the decrease induced by the rGIIA(D49S) was significant (10%). While the CCCP caused a two-fold increase in cell death compared with the untreated cells, the cell death caused by the rGIIA(D49S) was not statistically significant (results not shown). These results suggest that the effect of an excess of exogenous rGIIA on the mitochondria is too subtle to trigger the rapid death of a nerve cell.

### 2.7. rGIIA(D49S) Inhibited CCOX Activity in Rat Brain Tissue Sections

**Figure 6:**
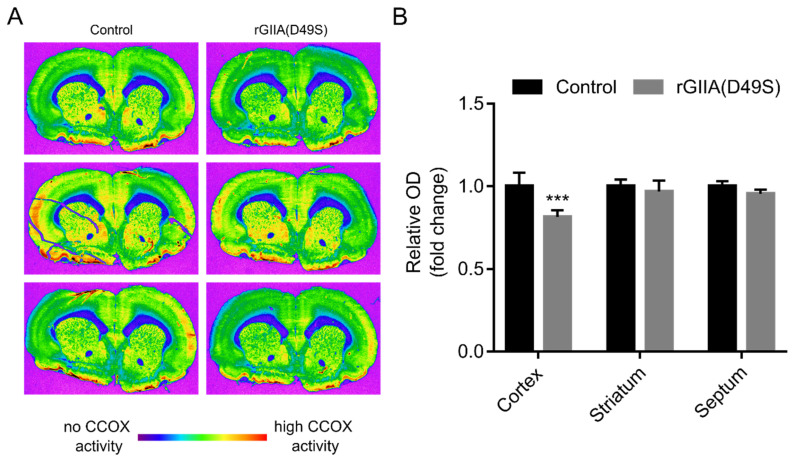
rGIIA(D49S) also affects the enzyme activity of CCOX ex vivo in tissue sections of rat brain. (**A**) Rats were sacrificed and coronal cryostat sections (10 µm) were cut through their rostral hippocampus. Consecutive sections were histochemically stained for CCOX activity in the presence or absence (control) of 10 µM rGIIA(D49S) and were imaged using a sensitive black-and-white video camera (see Materials and Methods section for details). Different shades of grey corresponding to different CCOX activity levels were visualized in the pseudo-color spectrum, using MCID M4 image-analysis software. Magnificaation 1.4×. (**B**) Densitometric analysis of the CCOX staining on cryosections from (A) in different ROIs: cerebral cortex, striatum and septum. Results are presented as means ± SD of fold change in relative optical density (ROD) compared to ROD of untreated sections from three independent experiments, and results that were statistically significantly different from the control are indicated (*** *p* < 0.001; two-way ANOVA with Bonferroni’s post-hoc test).

The involvement of GIIA in the pathogenesis of neurodegenerative diseases has already been suggested [8,9,10]. Having shown that exogenously supplied rGIIA molecules impair respiration in PC12 cells, which is potentially neurotoxic, we extended our experiments to the most relevant tissue associated with neurodegenerative diseases—rat brain tissue sections. The rats were sacrificed and slides with frozen sections of their brains, cut at the level of the striatum, were prepared using a cryostat. Consecutive sections were stained for CCOX activity in the presence or absence of 10 µM rGIIA(D49S). Again, only the enzymatically inactive mutant of rGIIA was tested for the reason stated in Section 2.5. The brain sections were imaged (Figure 6A) and analyzed for the relative optical density (ROD) corresponding to the CCOX activity in different brain regions of interest (ROI): the striatum, cerebral cortex and septum. The rGIIA(D49S) slightly but significantly decreased the CCOX activity in the cerebral cortex, while it had no statistically significant effect in the striatum and septum. However, a trend towards decreased CCOX activity in the presence of rGIIA(D49S) was also observed in these two regions. Potassium cyanide (KCN), a potent inhibitor of CCOX, was used as a positive control and as expected, it substantially inhibited CCOX activity in all the brain regions examined (not shown). These results strongly support the idea that an excess of exogenous rGIIA affects the neuronal cells by inhibiting their respiratory activity.

## 3. Discussion

GIIA plays both physiological and pathological roles in the mammalian brain. Physiologically, it is involved in the regulation of neurotransmission, neuritogenesis and mitochondrial homeostasis, and pathologically in cerebrovascular and neurodegenerative diseases [15,16]. The most prevalent neurodegenerative disorder for which there is still no cure is AD [17]. Although numerous studies have provided insights into the pathogenesis of this disease, the relative contribution of the underlying signaling and molecular pathways mediating the progressive cognitive decline remains largely unknown. Recently, AD was described as a multifactorial disease with mitochondrial dysfunction at the forefront [18,19]. According to the mitochondrial cascade hypothesis, these organelles are suggested to mediate or possibly even initiate pathological molecular cascades in AD. It has been reported that GIIA expression is increased in affected tissues [8] and that nerve cell death is preceded by the degeneration of the mitochondria [11]. Similar mitochondrial degeneration was reported when the nerve cells were exposed to snake venom β-neurotoxic GIIAs [12]. Since mammalian GIIAs and viperid β-neurotoxins are orthologous proteins, the observed effects might well share a common molecular mechanism. This is the case, for example, with the anticoagulant effect of human and some snake venom GIIAs, which in both cases is due to the inhibition of prothrombinase by binding to the activated blood coagulation factor X [20]. In this study, we therefore investigated whether the apparently similar effects of mammalian GIIA and Atx, a snake venom β-neurotoxic GIIA, on neuronal mitochondria have similar molecular backgrounds. This would qualify Atx as a molecular tool to better understand the role of GIIA in AD, and in particular to prove a causal link between an increased extracellular concentration of GIIA in the AD brains and the disease itself. 

PC12 cells have similar properties to neurons [21]. As shown previously, these cells are a suitable model cell line to study the neurotoxic effect of the snake venom Atx [13]. To compare the effects of the snake venom β-neurotoxin as credibly as possible with the effects of exogenously delivered mammalian GIIA, we also used this cell line in this study. Since PC12 cells are rat cells, we decided to perform our experiments with rat GIIA proteins, a wild-type rGIIA and its enzymatically inactive mutant, rGIIA(D49S). In GIIA, the aspartic acid residue in position 49 (D49) is crucial for the expression of enzymatic activity. If it is replaced by a serine, lysine, arginine, asparagine or glutamine residue, the protein becomes catalytically inactive [22]. As expected, the rGIIA showed normal phospholipase activity on the PyrPG vesicles, whereas the rGIIA(D49S) activity was undetectable. A single-point mutation hidden deep inside the protein molecule implies that the interactions of rGIIA(D49S) with other proteins remain very similar to those of rGIIA, as in the case of Atx and Atx(D49S) [13]. Indeed, both molecules were able to inhibit the formation of the specific adduct between ^125^I-Atx and its 25 kDa binding protein CCOX-II in mitochondrial membranes with a similar potency. 

rGIIA molecules could interfere with the formation of the adduct between ^125^I-Atx and CCOX-II by binding to the Atx binding site on the CCOX-II or to another binding site (protein) in close proximity. The latter is possible as the CCOX or respiratory chain complex IV consists of 13 different subunits [23]. Using rGIIA affinity resin, two protein bands were specifically enriched: one with an apparent molecular mass of 25 kDa and the other of 20 kDa. In the first band, CCOX-II was identified using MS/MS. rGIIA, as Atx, therefore physically interacts with CCOX-II. The identity of the 20 kDa rGIIA-binding protein remained unknown. However, the discrepancy between the large difference in affinity for CCOX-II between Atx and both rGIIA molecules, which was about two orders of magnitude (6 nM vs. 1 µM), and the comparable potencies of inhibition of CCOX activity in the isolated PC12 mitochondria (70% inhibition vs. 85% inhibition for Atx(D49S) and rGIIA(D49S), respectively), suggests that this protein is also part of the CCOX complex or endogenously regulates its activity. 

Like Atx(D49S), rGIIA(D49S) strongly inhibited the CCOX activity in isolated mitochondria. This activity of GIIA is obviously independent of its phospholipase activity, as is its cell internalization. Indeed, rGIIA(D49S) added to the medium containing PC12 cells passed through the plasma membrane and colocalized with mitochondria like Atx(D49S) [14]. The effect of rGIIA(D49S) on the cells in the culture was indicated by a slightly reduced TMRM fluorescence intensity, indicating a reduction in the MMP. The distortion induced by the rGIIA(D49S) was apparently not severe enough to cause a statistically significant cell death after 3 h of incubation. This is consistent with the observations that mitochondrial dysfunction occurs in the early stages of AD and likely precedes neuron loss [24,25], correlating with the nature of AD: a slowly progressive disease in which symptoms develop gradually over many years and become more severe over time [17]. 

To increase the relevance of the results obtained on neuron-like cells in a culture, we also tested the effect of rGIIA on its actual target tissue, the rat brain. As we were able to show, the phospholipase activity of the rGIIA was irrelevant for the inhibition of CCOX activity and for the cell internalization. So, to avoid ambiguous results due to the destruction of the gentle brain tissue by phospholipolysis, we incubated the brain sections with rGIIA(D49S). We found that rGIIA(D49S) slightly but significantly decreased the CCOX activity in the rat brain, especially in the cerebral cortex region. GIIA, which is overproduced and secreted by glial cells in AD tissue [16], can inhibit the respiratory activity of adjacent neuronal cells. Decreased CCOX activity and the inhibition of the respiratory chain were shown to increase the production of reactive oxygen species (ROS) [26,27], which in turn induced membrane lipid peroxidation, neuroinflammation and amyloid beta/tau aggregation [28,29]. This might alter the CCOX subunits and further impair its activity [30,31], resulting in a cycle that leads to neurodegeneration. Our conclusion is consistent with the results of a comprehensive proteome analysis of an AD brain and cerebrospinal fluid, reflecting changes in energy metabolism [32], as well as with the strong evidence for a decrease in CCOX activity in AD patients and animal models [33,34].

In summary, we characterized the effect of rGIIA on neuronal mitochondria and compared it with that of Atx, a β-neurotoxic snake venom GIIA. rGIIA and Atx bound to CCOX-II and interfered with each other. Both acted in a very similar manner on PC12 neuron-like cells. Independent of the phospholipase activity, rGIIA and Atx were internalized into the intact PC12 cells from the extracellular space and inhibited CCOX activity in the isolated mitochondria. In the intact PC12 cells, rGIIA(D49S) was able to decrease the TMRM fluorescence intensity, indicating a decrease in the MMP; however, it did not do so extensively enough to trigger cell death during the experiment. Most importantly, rGIIA also inhibited the CCOX activity in rat brains. GIIA has been linked to the aetiology of AD. By demonstrating that rGIIA and the snake venom β-neurotoxin Atx act very similarly on neuronal mitochondria, we qualified the latter as a promising molecular tool to study different aspects of GIIA in AD, its development, therapy and diagnosis.

## 4. Materials and Methods

### 4.1. Production and Characterization of the Recombinant Rat GIIA Proteins

The expression plasmid to produce the recombinant rGIIA was constructed by cloning the rGIIA gene, which is codon-optimized for bacterial expression, from pUC57 plasmid (GenScript, Piscataway, NJ, USA) into a previously constructed expression vector, which is based on pT7-7 [35]. For rGIIA(D49S), the mutation was introduced using the QuikChange II Site-Directed Mutagenesis Kit (Agilent, Santa Clara, CA, USA) and the primers 5′-GTGCTGCGTTACCCACAGCTGCTGCTACAACCGTC-3′ and 3′-CACGACGCAATGGGTGTCGACGACGATGTTGGCAG-5′ (desired mutations underlined), changing the Asp (D) residue at position 49 to the Ser (S) residue. rGIIA and rGIIA(D49S) were expressed in the form of inclusion bodies in *E. coli* BL21 (DE3). The in vitro refolding of the inclusion-body proteins proceeded as described in [14].

The recombinant proteins were analyzed using SDS-PAGE in the presence of 150 mM dithiothreitol on 15% (*m*/*v*) polyacrylamide gels. Their N-terminal sequences were determined with automated Edman degradation using a protein-sequencing system Procise 492A (Applied Biosystems, Waltham, MA, USA). A mass-spectrometry analysis of the products was performed using an electrospray ionization Q-TOF Premier mass spectrometer (Micromass, Wilmslow, UK). The enzymatic activity of the recombinant proteins towards PyrPG vesicles was performed as described in [36].

### 4.2. Heterologous Competition Assay

As previously described, the mitochondrial membranes containing P2d fraction was isolated from the porcine cerebral cortex [37] and was affinity-labeled with ^125^I-Atx [38]. In brief, 10 nM ^125^I-Atx and 0 nM, 400 nM, 800 nM, 1.2 µM, 1.6 µM, 1.9 µM, 2.2 µM, 2.5 µM, 5.0 µM, or 10.0 µM (all concentrations are final) of either unlabelled rGIIA or rGIIA(D49S) were added to the detergent extract of the membranes. In the case of the unlabeled Atx, concentrations of 0 nM, 2 nM, 7 nM, 10 nM, 20 nM, 50 nM, 100 nM and 200 nM were used. The reaction mixtures were incubated at room temperature for 30 min, and then reacted with a 100 µM (final concentration) cross-linker disuccinimidyl suberate (DSS; Pierce, Waltham, MA, USA). After 5 min, the cross-linking reaction was stopped with the addition of the reducing SDS-PAGE loading buffer. Samples were analyzed with SDS-PAGE, and the gels were dried and autoradiographed at −70 °C using Kodak X-Omat AR films [39]. The films were imaged using a ChemiDoc MP Imaging System (Bio-Rad, Hercules, CA, USA), the protein bands were quantified and the half-maximum inhibitory concentration (IC_50_) was calculated using a non-linear fit with Image Lab Software (Bio-Rad, Hercules, CA, USA).

### 4.3. Identification of rGIIA-Binding Protein in Porcine Cerebral Cortex Mitochondria

rGIIA was reacted with activated CHS at 1 mg per mL of swollen CHS gel, as described by Šribar et al. [40] to prepare the affinity resin rGIIA-CHS. A control resin, EA-CHS, was prepared in the same way, except that the reactive groups on the CHS were derivatized with ethanolamine (EA) instead of rGIIA. These derivatized gels were washed and equilibrated in 75 mM Hepes, pH 7.4, 150 mM NaCl, 2 mM CaCl_2_ and 0.2% (*m*/*v*) Triton X-100 (equilibration buffer). The P2d fraction detergent extract was prepared using 75 mM Hepes, pH 7.4, 150 mM NaCl, 2 mM CaCl_2_ and 2% (*m*/*v*) Triton X-100 (extraction buffer). Following a 10-fold dilution of the detergent extract with 75 mM Hepes, pH 7.4, 150 mM NaCl and 2 mM CaCl_2_, aliquots were incubated with rGIIA-CHS and EA-CHS overnight at 4 °C with gentle agitation. The gels were transferred to columns and washed with the equilibration buffer (20 volumes of the gel). The bound proteins were eluted from the gels by lowering the pH in two batches with 140 mM MES, pH 5.0, 200 mM NaCl, 4 mM CaCl_2_ and 0.2% (*m*/*v*) Triton X-100.

The affinity-purified proteins were analyzed with SDS-PAGE under reducing conditions (0.5% (*m*/*v*) SDS, 10% (*v/v*) glycerol, 50 mM DTT, 30 mM Tris/HCl, pH 6.8) and subsequent staining of the gels with colloid silver [41]. Parts of the gel containing protein bands were excised, de-stained [42] and processed for protein identification with mass spectrometry (MS), as described by [43]. For the protein identification, the obtained MS/MS spectra were checked against the SwissProt protein-sequence database, Taxonomy *Sus scrofa*.

### 4.4. Culturing of PC12 Cells

PC12 cells (ATCC CRL-1721, American Type Culture Collection, Manassas, VA, USA), a cell line derived from a pheochromocytoma of the rat adrenal medulla, were grown at 37 °C under 5% (*v/v*) CO_2_ with 10 cm culture plates in a F12K growth medium (Kaighn’s modification of Ham’s F-12; Gibco, Billings, MT, USA) containing 15% (*v/v*) horse serum, 2.5% (*v/v*) fetal bovine serum, 100 U/mL penicillin and 100 μg/mL streptomycin (i.e., the complete culture medium).

### 4.5. Isolation of Mitochondria from PC12 Cells and Measurement of CCOX Activity

Mitochondria were prepared from PC12 cells, as described in Ivanušec et al. [14], and the CCOX activity was measured using the Cytochrome c Oxidase Assay Kit (Sigma-Aldrich, St. Louis, MO, USA), as described previously [13]. Briefly, the isolated mitochondria (12 µg/mL total protein) were incubated for 30 min at room temperature in the presence of either 1 µM rGIIA or 1 µM rGIIA(D49S). The positive control contained mitochondria in the buffer A (10 mM Tris-HCl, pH 7.0, 120 mM KCl), while the negative control only contained the buffer A. Following incubation, the solution containing CytC_red_ was added to the final concentration of 22 µM and the decrease in absorbance at 550 nm (A_550_) of the reaction mixture was recorded on a microplate reader (Tecan, Männedorf, Switzerland) for 10 min, the time in which the CytC_red_ in the positive control was completely oxidized. Immediately after this, the absorption spectrum of the reaction mixture from 530 to 576 nm was measured on the same instrument. To determine the relative rate of CytC_red_ oxidation in the specified conditions, the average slope of the function y(t) = log [A_550_(t) − A_550_(600 s)] [44] in the first 400 s (linear part of the function) was calculated, the slope of the negative control was subtracted and the obtained result was normalized to the positive control. A_550_ (600 s) was obtained in the positive control at 600 s, when the CytC_red_ was completely oxidized. A statistical analysis was performed using Prism 7.0 (GraphPad Software, San Diego, CA, USA). The relative CytC_red_ oxidation rates are presented as means ± S.E.M. of the three independent experiments. Statistical significance was determined using a one-way ANOVA, followed by Tukey’s post-hoc test. *p* values less than 0.05 were considered statistically significant. 

### 4.6. Fluorescence Confocal Microscopy Colocalization Study on PC12 Cells

PC12 cells were plated on 4-well glass-bottom cell-culture plates (Greiner Bio-One, Frickenhausen, Germany) at a concentration of 4 × 10^4^ cells/well. Forty-eight hours after seeding, the cells were incubated in the presence of either 100 nM ^546^Alexa-rGIIA(D49S) or 100 nM ^546^Alexa-rGIIA for 3 h. The cells were then stained for mitochondria using MitoTracker™ Green FM (Thermo Fisher Scientific, Waltham, MA, USA) according to the manufacturer instructions, washed with the complete culture medium and imaged using an inverted confocal laser scanning microscope (Axio Observer Z1 LSM 710, ZEISS, Oberkochen, Germany) with a Plan-Apochromat 63/1.40 oil objective. Fluorophores were excited sequentially using argon (488 nm) and helium–neon (543 nm) lasers and the emitted light was collected through SP 545 and LP 545 filters. The mean degree of the colocalization of the red and green signals of at least 7 images was calculated using ZEISS ZEN software. Colocalization was presented as the Manders’ coefficient [45], i.e., the ratio of the summed intensities of the pixels from the green channel, for which the intensity in the red channel is above the threshold, to the total intensity in the green channel. An image of the colocalization was displayed using the “Colocalization” tab in the ZEISS ZEN software.

### 4.7. Measurement of TMRM Fluorescence Intensity and Cell Death

Cells were plated on 24-well cell-culture plates (TPP, Switzerland) at a concentration of 2 × 10^5^ cells/well, 3 days before performing an experiment and grown for 48 h in a complete culture medium, followed by 16 h of serum deprivation in an F12K growth medium. After starvation, the cells were incubated in the presence of either 1 µM rGIIA(D49S) or 100 µM of carbonyl cyanide 3-chlorophenylhydrazone (CCCP) for 3 h, and the uncoupler of the MMP was used as a positive control. Changes in the TMRM fluorescence intensity were determined using a modified TMRM-based assay described previously [46]. Floating and adherent cells were harvested, passed through a 22G hypodermic needle five times and centrifuged at 300× *g* for 10 min. The cell pellet was resuspended in 25 nM TMRM (Life Technologies, Carlsbad, CA, USA) in DPBS and incubated for 25 min in the dark at room temperature. To determine cell death, YO-PRO-1 iodide (Life Technologies, Carlsbad, CA, USA) was used in a final concentration of 50 nM and the cells were incubated for 10 min in the dark at room temperature. The cell suspension was diluted with 200 µL of DPBS containing 0.1% (*m*/*v*) fatty-acid-free bovine serum albumin (Sigma-Aldrich, St. Louis, MO, USA) and was analyzed by flow cytometry on a FACS Canto II system equipped with FL-1 (530/30) and FL-3 (650LP) filters. At least 2 × 10^4^ events were analyzed per sample. Statistical significance was determined using a one-way ANOVA, followed by Tukey’s post-hoc test. *p* values less than 0.05 were considered statistically significant.

### 4.8. Assessment of CCOX Activity in Rat Brain Tissue Sections

Young adult male Wistar rats were treated according to the Directive 2010/63/EU of the European Union and every effort was made to minimize their suffering. The euthanasia protocol was approved by the Republic of Slovenia’s Administration for Food Safety, Veterinary Sector and Plant Protection (Reference Number U34401-13/2021/4). The animals were sacrificed using CO_2_ inhalation followed by decapitation, and cryostat sections of their brains were prepared as described previously [47]. In brief, after decapitation, the brains were rapidly removed, quickly frozen on dry ice and stored at –20 °C until further use. Coronal sections (10 µm) were cut through the striatum (1 mm anterior from bregma; [48]) using a cryostat (Leica CM1950, Leica Biosystems, Wetzlar, Germany) and were thaw mounted onto glass slides coated with a 0.01% solution of poly-L-Lys. The sections were stored at –20 °C until further processing. Consecutive sections were then stained for CCOX activity following the diaminobenzidine (DAB) procedure [49,50] in the presence or absence of 10 µM rGIIA(D49S). The slides were transilluminated using a white-light transilluminator (Northern Light B90, Imaging Research Inc., St. Catharines, ON, Canada). Images of the brain sections were acquired using a sensitive black-and-white video camera (MTI, DAGE 72E, USA), and they were analyzed with ImageJ software [51] using Mean Gray Value measurements, from which the relative optical density (ROD) was calculated using the formula ROD(x) = log10(maximum gray value/x). To quantify the observed differences in CCOX staining between the treatment groups, the ROD was measured in the following regions of interest (ROIs): striatum, cerebral cortex and septum. We only considered the brain areas without damage or artifacts. For each section, the average ROD of an individual ROI was calculated from 1–3 bilateral measurements. The ROD of the *corpus callosum* (the region with presumably no CCOX activity) on the same section, which was used as the background ROD, was then subtracted from the ROD of each measurement. Finally, for different treatment groups, the average ROD value of each ROI was calculated from three independent sections. Statistical significance was determined using a two-way ANOVA, followed by Bonferroni’s post-hoc test. *p* values less than 0.05 were considered statistically significant.

## Data Availability

Data is contained within the article and Supplementary Material.

## Supplementary Materials

The following supporting information can be downloaded at: https://www.mdpi.com/article/10.3390/ijms232012368/s1.
