# Supplementary material for: Rat Group IIA Secreted Phospholipase A2 Binds to Cytochrome c Oxidase and Inhibits Its Activity: A Possible Episode in the Development of Alzheimer’s Disease
Source: Int J Mol Sci. 2022 Oct 15;23(20):12368. doi: 10.3390/ijms232012368 (PMC9604285; doi:10.3390/ijms232012368)
Supplement: Supplementary file 1 [file ijms-23-12368-s001.zip › ijms-1933684-supplementary.pdf]

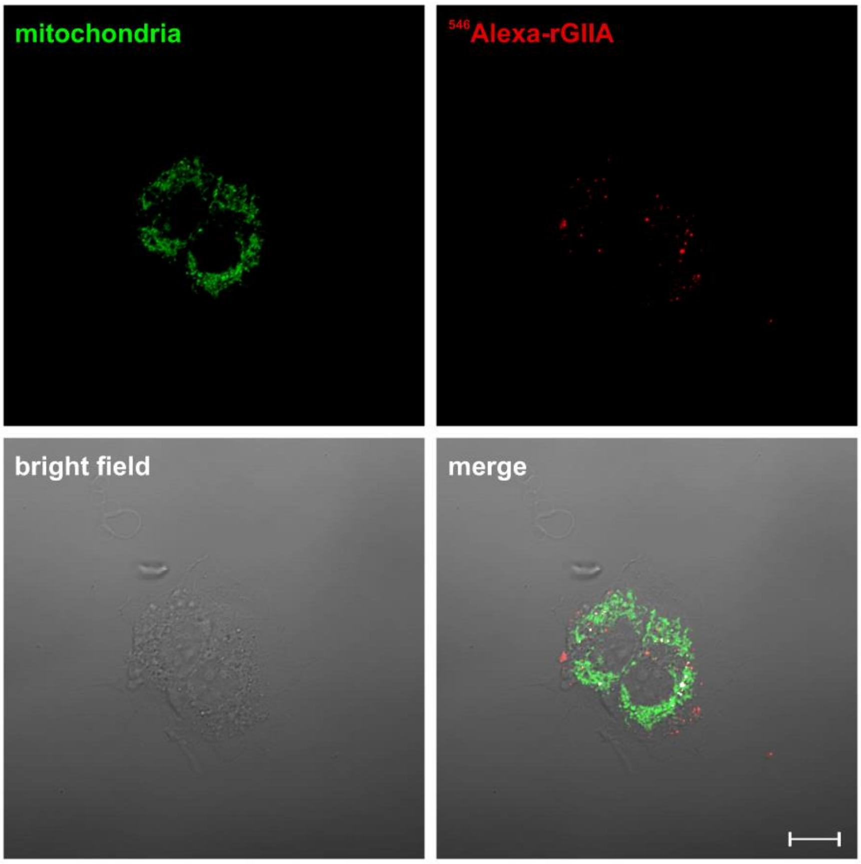

Figure S1. rGIIA can reach mitochondria in PC12 cells from the extracellular space. PC12 cells were incubated with 100 nM ^{546}Alexa-rGIIA for 3 h and mitochondria labelled with MitoTracker Green FM dye. Cells were then analysed with confocal microscopy. Representative confocal fluorescence microscopy image shows ^{546}Alexa-rGIIA (red) and MitoTracker-labelled mitochondria (green); colocalization in merged image is shown in white. The extent of colocalization of ^{546}Alexa-rGIIA and mitochondria in PC12 cells, expressed in terms of the Manders' coefficient, was 0.04 ± 0.01, mean ± S.E.M., as calculated from 10 images. Scale bar, 10 μm.
